# World species of the subgenus *Oligonychus* (*Reckiella*) Tuttle and Baker (Acari, Prostigmata, Tetranychidae), diagnostic keys, taxonomic notes, and a new species

**DOI:** 10.1038/s41598-023-40436-7

**Published:** 2023-08-17

**Authors:** Hafiz Muhammad Saqib Mushtaq, Muhammad Kamran, Fahad Jaber Alatawi

**Affiliations:** Acarology Laboratory, Department of Plant Protection, College of Food and Agriculture Sciences, King Saud University, P.O. Box No. 2460, 11451 Riyadh, Saudi Arabia

**Keywords:** Evolution, Zoology

## Abstract

*Oligonychus* Berlese (Acari, Prostigmata, Tetranychidae) is an agriculturally important and the largest genus of spider mites, comprised of 211 species (including new species), two subgenera, four species groups, and 11 species subgroups. The present study comprehensively addressed the morphotaxonomic-based identification of world species of the subgenus *Reckiella* Tuttle and Baker. Five diagnostic keys were developed for identifying *Oligonychus* (*Reckiella*) species belonging to five species subgroups: *iseilemae*, *pritchardi*, *biharensis*, *gossypii*, and *exsiccator*. Taxonomic notes are provided on intraspecific variations and some closely related *Oligonychus* (*Reckiella*) species representing six species complexes, viz. the *afrasiaticus* complex, the *litchii* complex, the *pratensis* complex, the *plegas* complex, the *sacchari* complex, and the *tylus* complex. One new spider mite species, *Oligonychus bahaensis* sp. nov., is described and illustrated from Grasses (Poaceae) under the subgenus *Reckiella*.

## Introduction

Spider mites belonging to the genus *Oligonychus* Berlese (Acari, Prostigmata, Tetranychidae) are economically important pests of various agronomic crops^1,2^. Such as the date palm mite *O. afrasiaticus* (McGregor), the tea red spider mite *O. coffeae* (Neitner), and the banks grass mite *O. pratensis* (Banks) are considered key agricultural pests^3,4^.

The *Oligonychus* is the largest genus in the family Tetranychidae Donnadieu, consisting of two subgenera: *Oligonychus* Berlese and *Reckiella* Tuttle and Baker. It comprises 210 spider mite species^2,5,6^, excluding two recently synonymized species, viz. *O. mangiferus* (Rahman and Sapra) and *O. vitis* Zaher and Shehata^7^. However, the subgenus *Reckiella* contains 119 species^5,6^.

*Oligonychus* species are very challenging to identify due to morphological similarities in females^4^, limited numbers of potential diagnostic characters, and intraspecific variations^5,8^. Furthermore, precise differentiation among *Oligonychus* species must have specimens of both sexes^5,8,9^. Moreover, most *Oligonychus* species have only been accurately identified based on differences in shape and orientation of male aedeagus^5,8,10^. In addition, various species complexes have been highlighted in this genus^4,5,7,8,10–12^. Such complexes require a comprehensive taxonomic revision to be resolved^7^. Indeed, no species-level diagnostic key has yet been developed to identify world *Oligonychus* species, except for some regionally prepared keys^8,10,11,13–17^. However, a world diagnostic key was constructed to identify subgenera, species groups, and species subgroups of the genus *Oligonychus*^5^.

This study provides five diagnostic keys to the world *Oligonychus* species of the subgenus *Reckiella* with taxonomic notes on intraspecific variations and some closely related *Oligonychus* (*Reckiella*) species. In addition, one new species *O. bahaensis* sp. nov. is described under the subgenus *Reckiella.*

## Methodology

All the published taxonomic literature of world *Oligonychus* species belonging to the subgenus *Reckiella* was collected through personal communication with acarologists in different countries, using various search engines/websites as well as the websites of different acarological/entomological research journals. The published *Oligonychus* literature, i.e., original and subsequent descriptions of all species and regionally prepared diagnostic keys, was critically reviewed to develop a key for identifying *Oligonychus* (*Reckiella*) species.

**Figure 1:**
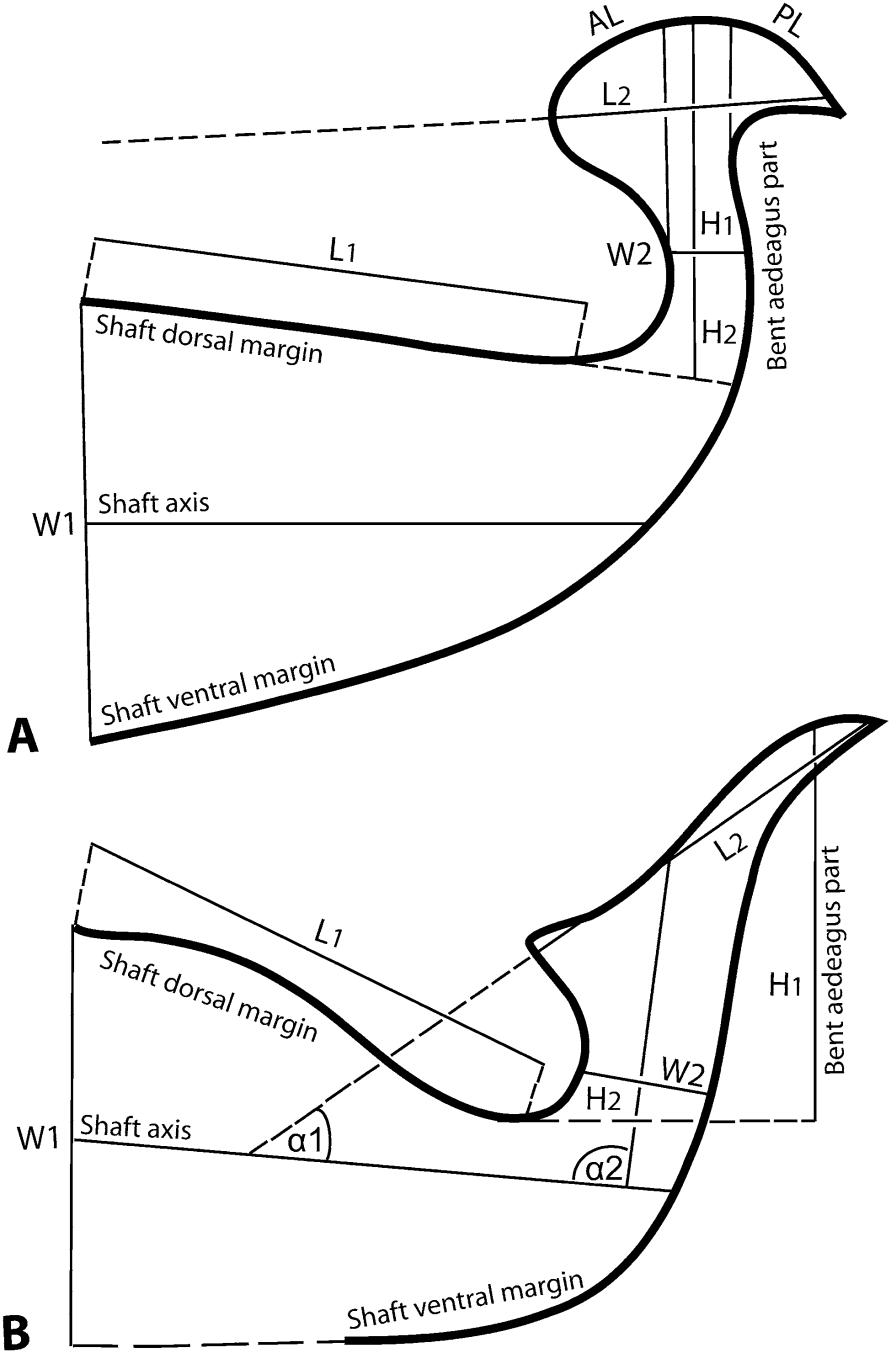
Morphological aedeagal parameters (H_1_: height of bent aedegal part, measured from the level when shaft dorsal margin just starting to bend upward (**A** either shaft dorsal margin straight, e.g. in *Oligonychus afrasiaticus* or **B** undulating, e.g. in *O. oenotherae* Smiley and Baker) to maximum height of knob dorsal margin, H_2_: height of knob stem, measured from the level when shaft dorsal margin just starting to bend upward to the level of measuring stem width, L_1_: length of shaft dorsal margin; L_2_: length of knob, W1: width of shaft, W2: width of knob stem, AL: length of knob anterior projection, PL: length of knob posterior projection, α1: angle formed between shaft axis and knob axis, and, *α2:* angle formed between shaft axis and bent part axis) that measured and used in the diagnostic keys for differentiation among some *Oligonychus* species of the subgenus *Reckiella*.

Specimens of the new species (*O. bahaensis* sp. nov.) described in the present study were collected in 2019/2021 from grasses (Poaceae) in the Baha province of Saudi Arabia (SA). A sample of *O. pratensis* was also received from the country (USA) of its type locality, which was sent by our colleagues^18^ at our request. Slide-mounted male and female specimens were observed under a BX51 phase-contrast microscope (Olympus, Tokyo, Japan), and the generic level identification was performed following the key of Bolland, et al.^3^. Whereas the subgenera, species groups, and subgroups were identified using the diagnostic key of Mushtaq et al.^5^, and species-level identification was performed following the key developed in the present study. The nomenclature of Lindquist^19^ was followed for leg chaetotaxy and other terminologies, and Grandjean^20^ and Grandjean^21^ for body setae. Furthermore, following Mushtaq et al.^6^, different traits of the male aedeagus were measured to identify new species and for differentiation among closely related species belonging to the *Reckiella* (Fig. 1). All measurements for new species are provided in micrometers. First, the sizes are given for the holotype, followed by the range in parenthesis for paratypes. The collected mite specimens, including the holotype and paratypes, were deposited at the King Saud University Museum of Arthropods (Acarology section), Department of Plant Protection, College of Food and Agriculture Sciences, King Saud University (KSU), Riyadh, SA.

## Results

**Family** Tetranychidae Donnadieu.

**Subfamily** Tetranychinae Berlese.

**Tribe** Tetranychini Reck.

**Genus** *Oligonychus* Berlese.

**Type species** *Heteronychus brevipodus* Targioni-Tozzetti, 1878: 255.

**Diagnosis** (based on female and male). As defined by Mushtaq, et al.^5^.

**Subgenus** *Reckiella* Tuttle and Baker.

**Type species** *Tetranychus pratensis* Banks, 1912: 97.

**Diagnosis** (based on male). As defined by Mushtaq, et al.^5^.

### New species

#### *Oligonychus bahaensis* sp. nov.

*Diagnosis* (based on male and female)—Male: Empodium I with two claws, proximoventral claw subequal or slightly shorter than main dorsal claw; aedeagus turns dorsad with distinct knob, knob with narrowly rounded anterior projection and acute posterior projection, shaft axis forming strong acute angle with knob axis. Female: Tibia I with nine and tibia II with seven tactile setae; peritremes straight, with bulbous end. Opisthosoma medially with transverse striae, except longitudinal/irregular longitudinal or slightly oblique striae with/without forming V-shaped pattern in-between setae *f*_*1*_ and *f*_*2*_, dorsal setae slender, serrate, not set on tubercles and longer than longitudinal distances to the bases of setae next in-line.

**Figure 2:**
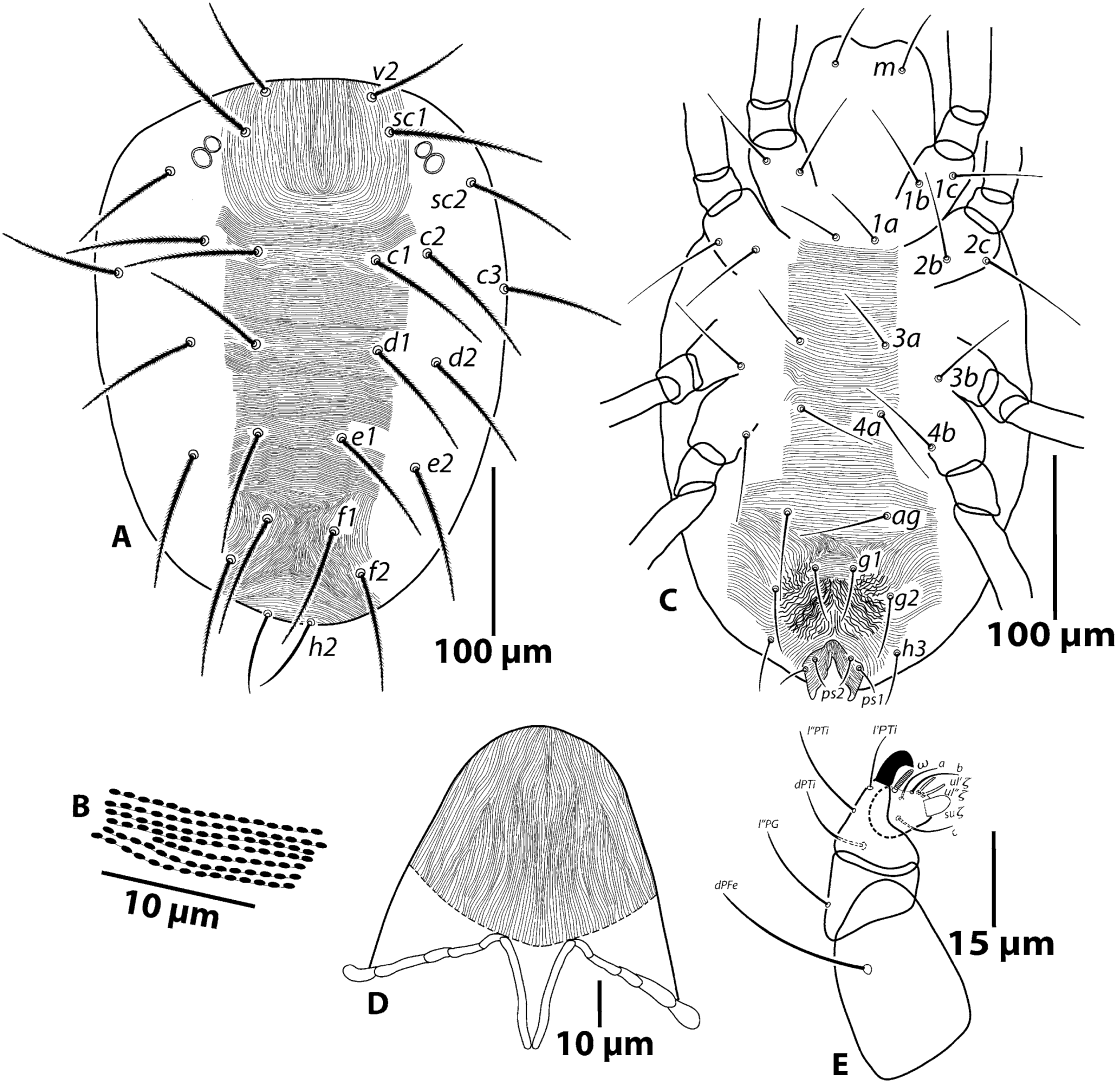
*Oligonychus bahaensis* sp. nov., Female: (**A**) dorsum, (**B**) lobes on dorsal striae, (**C**) venter, (**D**) peritreme and stylophore, (** E**)—palp.

**Figure 3:**
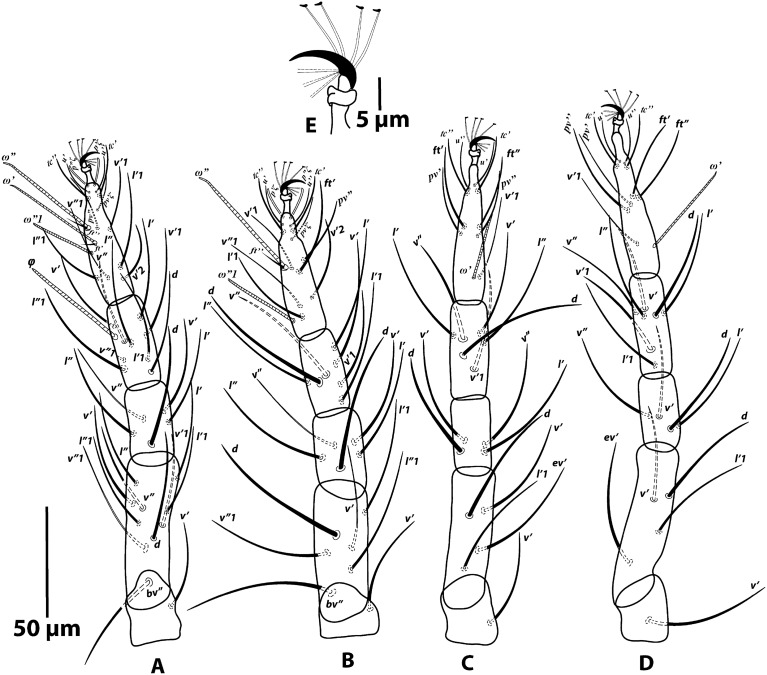
*Oligonychus bahaensis* sp. nov., Female: (**A**) leg I (left), (**B**) leg II (left), (**C**) leg III (left), (**D**) leg IV (left), (**E**) empodium IV (left).

*Female* (Figs. 2 and 3; n = 5)—Length of body (*v*_*2*_–*h*_*2*_) 310 (282–310); width (*c*_*3*_–*c*_*3*_) 225 (221–225).

*Dorsum* (Figs. 2A,B): Propodosoma medially with longitudinal striae; opisthosoma medially with transverse striae except area in-between setae *f*_*1*_ and *f*_*2*_ with distinct patch of longitudinal/irregular longitudinal or slightly oblique striae with/without forming V-shaped pattern; lateral idiosoma with longitudinal to oblique striae; dorsal striae with small, semicircular lobes, wider than long (Fig. 2B); dorsal setae slender, serrate, not set on tubercles and longer than longitudinal distances to the bases of setae next in-line (Fig. 2A). Lengths of dorsal setae *v*_*2*_ 61 (61–63), *sc*_*1*_ 92 (84–92), *sc*_*2*_ 67 (67–72), *c*_*1*_ 79 (79–81), *c*_*2*_ 82 (74–82), *c*_*3*_ 72 (72–79), *d*_*1*_ 75 (74–75), *d*_*2*_ 75 (75–80), *e*_*1*_ 74 (74–80), *e*_*2*_ 82 (75–82), *f*_*1*_ 74 (74–76), *f*_*2*_ 71 (69–71), *h*_*2*_ 46 (43–46). Distances between dorsal setae: *v*_*2*_*–v*_*2*_ 62 (59–62)*, sc*_*1*_–*sc*_*1*_ 83 (79–83), *sc*_*2*_*–sc*_*2*_ 172 (172–175), *c*_*1*_*–c*_*1*_ 69 (67–69), *d*_*1*_–*d*_*1*_ 71 (71–75), *e*_*1*_*–e*_*1*_ 48 (47–49), *f*_*1*_*–f*_*1*_ 39 (32–39), *h*_*2*_*–h*_*2*_ 26 (26–31), *c*_*1*_*–d*_*1*_ 51 (49–51), *d*_*1*_*–e*_*1*_ 51 (46–51), *e*_*1*_*–f*_*1*_ 49 (49–53), *f*_*1*_*–h*_*2*_ 54 (51–54).

*Venter* (Fig. 2C): Coxisternal area between coxae I–II and area between setae *1a*–*ag* with smooth, transverse striae; lateral opisthosoma with longitudinal to oblique lobed/smooth striae, pregenital region with irregular longitudinal to oblique striae, striae on genital flap transverse. Lengths of setae *1a* 36 (35–37), *1b* 51 (50–52), *1c* 52 (52–56), *2b* 52 (50–52), *2c* 67 (62–67), *3a* 38 (36–38), *3b* 54 (50–54), *4a* 46 (45–48), *4b* 51 (51–53), *ag* 52 (50–52), *g*_*1*_ 34 (34–36), *g*_*2*_ 38 (37–38), *ps*_*1*_ 16 (16–17), *ps*_*2*_ 16 (15–16), *h*_*3*_ 33 (29–33).

*Gnathosoma* (Fig. 2D–E): Ventral infracapitulum with seta *m* 38 (38–43). Palp spinneret *su*ξ length 4.5 (4–4.5), width 2.8 (2.4–2.7), solenidion ω length 4 (4–5) (Fig. 2E). Stylophore rounded. Peritremes simple, straight and oval-shaped or bulbous distally (Fig. 2D).

*Legs* (Fig. 3A–E): Length of legs (excluding coxae) leg I 247 (231–247), leg II 195 (187–195), leg III 220 (205–220), leg IV 251 (230–251). Leg segment setal formula as follows: coxae 2-2-1-1; trochanters 1-1-1-1; femora 10-6-4-4; genua 5-5-4-4; tibiae 9/8(1φ)-7-6-7; tarsi 13(1ω + 2dup)*-*13(1ω + 1dup)-9(1ω)-9(1ω). Tarsus I with four tactile setae proximal to proximal duplex setae (Fig. 3A) and tarsus II with three tactile setae proximal to duplex seta (Fig. 3B). Variation in leg setal counts for the 20 specimens are summarized as follows: tibia I with 9 tactile setae (n = 5), 8/9 setae (n = 9) and 8 setae (n = 6). Empodia claw like with three pairs of proximoventral hairs (Fig. 3E).

**Figure 4:**
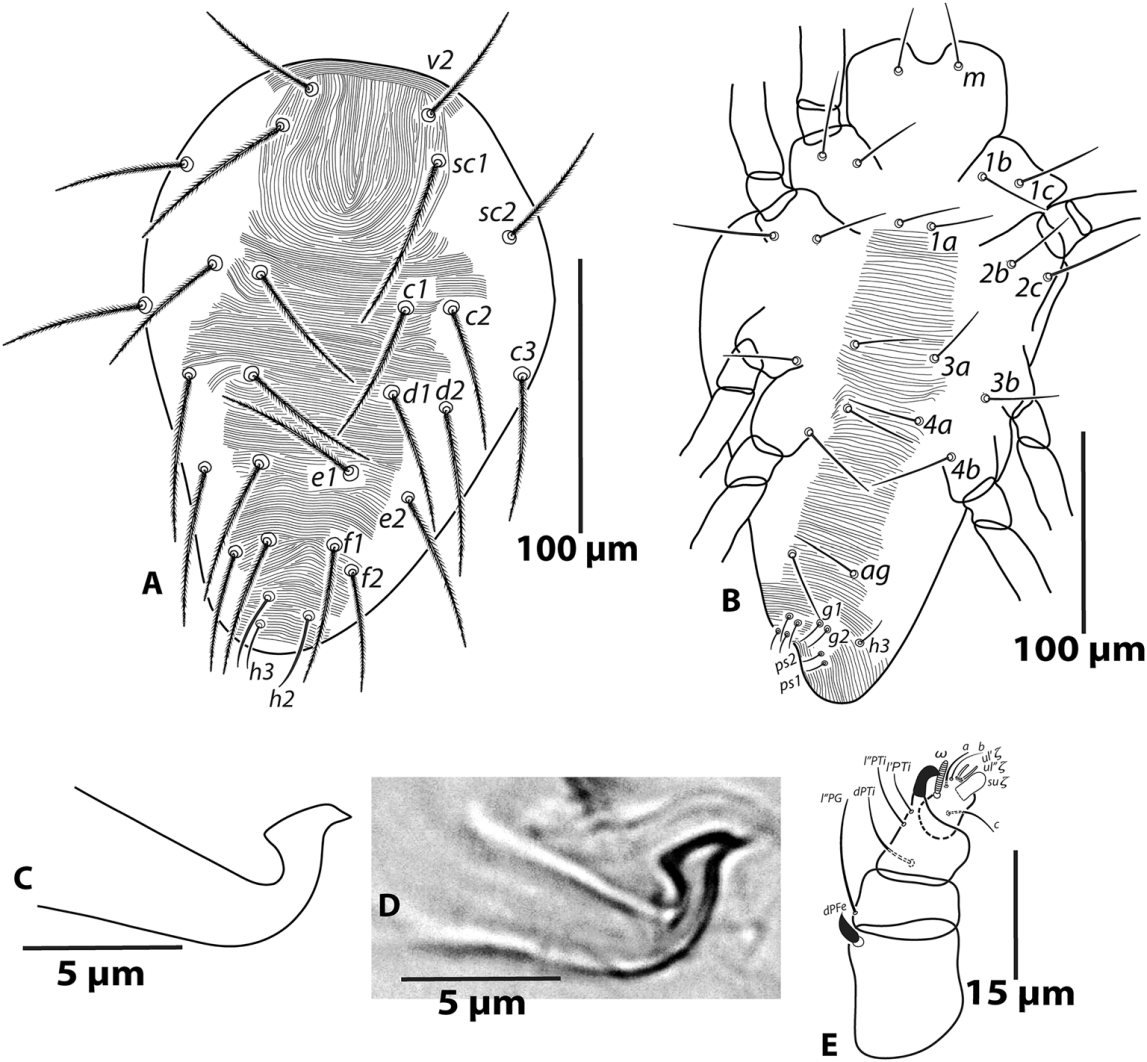
*Oligonychus bahaensis* sp. nov., Male: (**A**) dorsum, (**B**) venter, (**C**), (**D**) aedeagus, (**E**)—palp.

**Figure 5:**
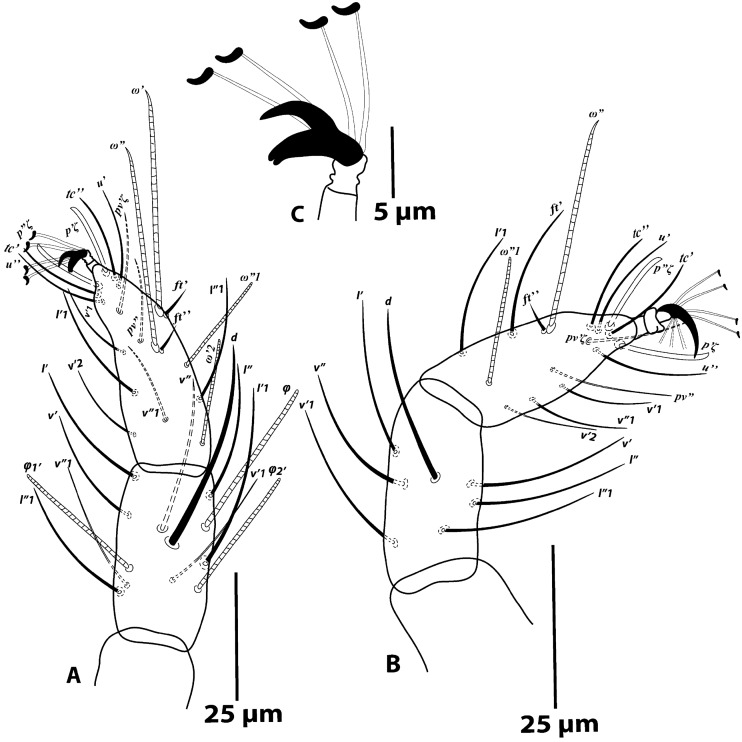
*Oligonychus bahaensis* sp. nov., Male: (**A**) tibia and tarsus I, (**B**) tibia and tarsus II, (**C**) empodium I.

*Male* (Figs. 4 and 5; n = 1): Length of body (*v*_*2*_–*h*_*2*_) 187; width (*c3*–*c3*) 140.

*Dorsum* (Fig. 4A): Propodosoma medially with longitudinal striae; opisthosoma medially and posteriorly with typical transverse striae or irregular transverse striae, forming weak inverted V-pattern in-between setae *f*_*1*_ and *f*_*2*_; lateral idiosoma with longitudinal to oblique striae; all striae without lobes. Dorsal setae (except *h*_*2*_ and *h*_*3*_) slender, serrate, not set on tubercles and longer than longitudinal distances to the bases of setae next in-line. Lengths of dorsal setae: *v*_*2*_ 47, *sc*_*1*_ 65, *sc*_*2*_ 48, *c*_*1*_ 52, *c*_*2*_ 51, *c*_*3*_ 53, *d*_*1*_ 52, *d*_*2*_ 62, *e*_*1*_ 55, *e*_*2*_ 60, *f*_*1*_ 52, *f*_*2*_ 45, *h*_*2*_ 26. Distances between dorsal setae: *v*_*2*_*–v*_*2*_ 46*, sc*_*1*_–*sc*_*1*_ 53, *sc*_*2*_*–sc*_*2*_ 120, *c*_*1*_*–c*_*1*_ 54, *d*_*1*_–*d*_*1*_ 53, *e*_*1*_*–e*_*1*_ 33, *f*_*1*_*–f*_*1*_ 25, *h*_*2*_*–h*_*2*_ 16, *c*_*1*_*–d*_*1*_ 37, *d*_*1*_*–e*_*1*_ 33, *e*_*1*_*–f*_*1*_ 28, *f*_*1*_*–h*_*2*_ 30.

*Venter* (Fig. 4B): Ventral integument with transverse striae, without lobes. Lengths of setae *1a* 30, *1b* 35, *1c* 42, *2b* 36, *2c* 49, *3a* 31, *3b* 36, *4a* 36, *4b* 40, *ag* 35, *g*_*1*_ 13, *g*_*2*_ 13, *ps*_*1*_ 10, *ps*_*2*_ 10, *h*_*3*_ 16.

*Aedeagus* (Fig. 4C,D; n = 3): Dorsally bent portion with distinct knob, knob with narrowly rounded anterior projection and acute posterior projection, directed dorsocaudad/caudad but not ventrad; knob dorsal margin slightly convex with inward bending near bases of posterior projection; posterior projection equal or subequal than anterior projection and stem width/height, knob length about 2.5 times or more than the stem-width and subequal or more than three times the stem-height; shaft axis forming strongly acute angle with knob axis and about right or weakly acute angle with axis of upturned part; shaft dorsal margin about 1.5 times or more longer than shaft maximum width and about twice or more longer than knob length; shaft dorsal margin forming an angle to shaft axis.

*Gnathosoma* (Fig. 4E): Ventral infracapitulum with seta *m* 31. Palp spinneret *su*ξ length 3 (3–4), width 2 (2–2.1), solenidion ω length 4 (4–5) (Fig. 4E). Stylophore rounded. Peritremes simple, straight and very slightly bulbous distally (similar to female).

*Legs* (Fig. 5A–C): Length of legs (excluding coxae) leg I 176, leg II 133, leg III 163, leg IV 167. Legs segments setal formula as follows: coxae 2-2-1-1; trochanters 1-1-1-1; femora 10-6-4-4; genua 5-5-4-4; tibiae 8/9(2/3φ)-7-6-7; tarsi 13(2ω + 2dup)*-*13(1ω + 1dup)-9(1ω)-9/10(1ω). Tarsus I with four tactile setae proximal to proximal duplex setae (Fig. 5A) and tarsus II with three or four tactile setae proximal to duplex seta (Fig. 5B). Empodium I with 2 claws, ventral proximoventral claw distally split as bifid, subequal or slightly shorter than free part of dorsal claw (Fig. 5C); empodial claws II–IV sickle-shaped with 3 pair of proximoventral hairs (similar to female).

*Etymology:* The specific epithet *bahaensis* was derived from the name of the province (Baha) of Saudi Arabia, from which the new species was collected.

*Type material:* Holotype male and paratypes twenty females and four males, grasses (Poaceae), Bani Saad, Baha province, November/September 2019/2021, 20° 00′ 39.4″ N, 41° 25′ 34.7″ E, coll. HMS Mushtaq M Kamran, EM Khan, and N Elgoni.

**Figure 6:**
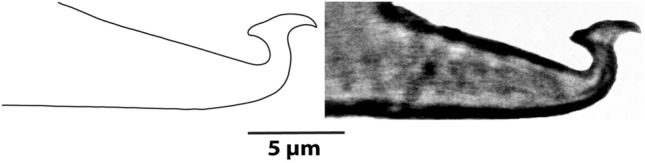
*Oligonychus pratensis* (Banks), Male: aedeagus.

*Remarks: Oligonychus bahaensis.* sp. nov. closely resembles *O. pratensis* (Banks), because both have female palp spinneret maximum two times longer than its width, only male empodium I with proximoventral claw, female/male peritremes straight with normal length, not hooked distally, female tibia I with 9 and II with 7 tactile setae, and various similar aedeagal characteristics, e.g., aedeagus with the distinct terminal knob, knob with rounded anterior and acute posterior projections, the axis of aedeagal knob forming an acute angle with the shaft axis. However, the new species differs from *O. pratensis* by male aedeagus knob with posterior projection about sub-equal or slightly longer than anterior projection (vs. 1.5 times longer), shaft dorsal margin maximum 2.5 the length of knob (vs. about three times longer) in *O. pratensis* (Fig. 6). Additionally, a high genetic divergence of 17.7% (ITS2) was observed between *O. bahaensis* sp. nov. (GenBank accession number: OR288175) and *O. pratensis* (GenBank accession number: OQ185450)^22^, aligned with the previous findings of interspecific divergences among various tetranychid species^7,23^.

Although we comprehensively explored the spider mites’ fauna from numerous localities/hosts in almost the whole of SA since 2018^5,7,22,24^, this new species *O. bahaensis* sp. nov. is observed to inhabit grasses in only one locality in Baha province.

### Keys to the world *Oligonychus* species of the subgenus *Reckiella* (114 species, excluding *O. comptus* and five briefly described species)

#### Key to species of the subgroup iseilemae (12 species)

**Table:** 

1. Distal part of dorsally bent aedeagal portion narrower than basal part, tapering distally or forming a slender hook, without barbs……………………………………………………***O. iseilemae*** (Hirst)	Distal part (knob/head) of dorsally bent aedeagal portion wider than basal part (stem/neck), without tapering distally………………………………………………………………………**2**
2. Posterior projection of aedeagal knob longer than neck width………………………………………. **3**	Posterior projection of aedeagal knob subequal or shorter than neck width………………………………… **5**
3. Aedeagal knob without anterior projection, but with rounded posterior projection, not tapering distally; female with 4 and 3 tactile setae proximal to duplex on tarsus I and II, respectively; female with v-shaped striae pattern between setae *e*_*1*_*-e*_*1*_…***O. megandrosoma*** Flechtmann and Alves	Aedeagal knob with clear acute anterior and posterior projections, later tapering distally; female with 3 and 1/2 tactile setae proximal to duplex on tarsus I and II, respectively; female with transverse striae pattern between setae *e*_*1*_*-e*_*1*_…………………………………………………………………… **4**
4. Female with 1 tactile seta proximal to duplex on tarsus II and empodium I with 4 pairs of proximoventral hairs; posterior projection of aedeagal knob gradually narrows toward tip, with almost straight dorsal and ventral margins; dorsal margin of shaft bent upward with strong curvature forming a clear stem; shaft axis at 105° angle (*α2*) with stem axis…………………………………………………… ***O. fileno*** Mendonca, Navia and Flechtmann	Female with 2 tactile setae proximal to duplex on tarsus II and empodium I with 3 pairs of proximoventral hairs; posterior projection of aedeagal knob abruptly narrows toward tip; dorsal margin of shaft bent upward with weak curvature forming a weak stem; shaft axis at 121° angle (*α2*) with stem axis………………***O. occidentalis*** Gutierrez
5. Axis of aedeagal knob parallel or subparallel to shaft axis, without forming an angle (*α1*) ………… **6**	Axis of aedeagal knob forming an angle (*α1*) with shaft axis……………………………………….. **7**
6. Aedeagal knob with prominent anterior and posterior projections; anterior projection acute, posterior projection indented medially; shaft axis at 108° angle (*α2*) with stem axis………………………………………………… ***O. beeri*** Estebanes and Baker	Aedeagal knob without obvious anterior and weak posterior projections; posterior projection acute without medial indentation; shaft axis at 125° angle (*α2*) with stem axis…………………………………***O. chiapensis*** Estebanes and Baker
7. Dorsal margin of aedeagal shaft abruptly upturned forming almost acute angle with anterior margin of stem; both anterior and posterior projections of aedeagal knob sub-equal or equal in length……. **8**	Dorsal margin of aedeagal shaft gradually upturned forming almost obtuse angle with anterior margin of stem; posterior projection of aedeagal knob clearly longer than anterior one (if present)……** 9**
8. Aedeagal knob very small, about one-fourth to one-fifth the length of dorsal margin of shaft…………………………………………… ***O. amnicolus*** Meyer	Aedeagal knob comparatively large, about one-half the length of dorsal margin of shaft………………………………………………………… ***O. themedae*** Meyer
9. Dorsal margin of aedeagal knob straight/smooth…………………………………………………..** 10**	Dorsal margin of aedeagal knob slightly wavy/convex or concave….........**11**
10. Dorsal margin of aedeagal shaft slightly bends internally; shaft axis forming almost 20^°^ angle (*α1*) with knob axis………………………………………….. ***O. anonae*** Paschoal	Dorsal margin of aedeagal shaft straight, without internal bends; shaft axis forming almost 35^°^ angle (*α1*) ………………………………***O. bagdasariani*** Baker and Pritchard
11. Dorsal margin of aedeagal knob slightly concave, proximal to anterior projection; terminal part of posterior projection bent downward………***O.poutericola*** Feres and Flechtmann	Dorsal margin of aedeagal knob convex, proximal to anterior projection; terminal part of posterior projection directed dorso-caudad…………………… ***O. acugni*** (Livshits)

### *Key to species of the subgroup *pritchardi* (13 species)*

**Table:** 

1. Male tibia I with 5 tactile setae (proximal member of duplex setae on tarsus I and II long, 1/2 to 1/3 as long as distal member)…………………………………………………………………… ***O. quercus*** Tuttle et al	Male tibia I with 8–10 tactile setae……………………………………… **2**
2. Dorsally bent part of aedeagus elongated, tapers gradually to an acute dorsally directed tip; without forming a knob………………………… ***O.longipenis*** Feres and Flechtmann	Dorsally bent part of aedeagus forming a clear knob or bending ventrad distally…………………… **3**
3. Aedeagus shaft first upturned then almost distal one-half bends ventrad, distal part gradually tapering; without forming knob/anterior and posterior projections distally……………………………… **4**	Aedeagus shaft with distinct anterior and posterior projection forming knob……………………….. **5**
4. Female with 3 tactile setae proximal to duplex on tarsus II; male with 10 tactile setae and 2 solenidion on tibia I; aedeagal shaft first slightly upturned, then gradually bends ventrad with strong round curve, terminal part directed anteriorly…………………… ***O. psidii*** Flechtmann	Female with 2 tactile setae proximal to duplex on tarsus II; male with 9 tactile and 3 sensory setae on tibia I; aedeagal shaft first loosely upturned, then suddenly bends ventrad at almost a right angle…………………………………… ***O. psidium*** Estebanes and Baker
5. Aedeagal knob with both anterior and posterior projections small; posterior projection not bending downward………………………………… **6**	Aedeagal knob with anterior projection very small, posterior projection very large; posterior projection tapering gradually and/or bending ventrad…………………… **7**
6. Femur II with 6 setae both in male and female; aedeagal knob with clear rounded anterior projection, dorsal margin of shaft first suddenly bends ventrad then dorsad forming a strong curve but weak stem………………………………………… ***O. festucolus*** Beard and Walter	Femur II with 7 setae both in male and female; aedeagal knob with inconspicuous/ very small angulated anterior projection, dorsal margin of shaft gradually bends dorsad forming a strong stem………………………………… ***O. tiwakae*** Gutierrez
7. Female with 2 tactile setae proximal to proximal duplex on tarsus I.… ***O. calcis*** Baker and Pritchard	Female with 3–4 tactile setae proximal to proximal duplex on tarsus I………………**8**
8. Aedeagal knob with terminal downturned part of posterior projection nearly/clearly reaching at the level of shaft axis or ventral margin of shaft…………………………… **9**	Aedeagal knob with terminal downturned part of posterior projection not reaching at the level of shaft axis…………………………………………… **10**
9. Aedeagus with terminal knob slightly angulate on dorsal side, posterior projection almost straight or slightly downturned, terminally………………………………***O. pritchardi*** (McGregor)	Aedeagus with distal knob strongly curved on dorsal side, posterior projection bends ventrad, terminally………………………… ***O. propetes*** Pritchard and Baker
10. Aedeagal knob with 1/2 terminal part of posterior projection suddenly bends ventrad (anterior projection sharp/acute) …………………………………………………….. ***O. flechtmani*** Tuttle et al	Aedeagal knob with posterior projection curved gradually, terminal part nearly straight or downturned…………………………………………**11**
11. Female with 3 and 1 tactile setae proximal to duplex on tarsus I and II, respectively; male with 6 or 7 tactile setae on tibia II; aedeagal knob with prominent rounded anterior projection……………………………………***O. quasipropetes*** Flechtmann	Female with 4 and 3 tactile setae proximal to duplex on tarsus I and II, respectively; male with 5 tactile setae on tibia II; aedeagal knob with inconspicuous/slight acute anterior projection……….… **12**
12. Male tarsus IV with very short solenidion…………………………. ***O. mimosae*** Baker and Pritchard	Male tarsus IV with very long solenidion……………… ***O. veranerae*** Baker and Pritchard

#### *Key to species of the subgroup *biharensis* (10 species)*

**Table:** 

1. Female peritreme hooked or U-shaped distally………………………… **2**	Female peritreme straight distally………………………………… **5**
2. Aedeagal knob with posterior projection gradually bending ventrad, tip almost reaching the level of shaft dorsal margin………..…………………………***O. biharensis*** (Hirst)	Aedeagal knob with posterior projection tip directed dorsad or caudad, tip not reaching the level of shaft dorsal margin ……………………………………… **3**
3. Aedeagal knob with dorsal margin concave or slightly sigmoid………………. ***O. antherus*** Rimando	Aedeagal knob with dorsal margin convex or straight………………………… **4**
4. Aedeagal shaft with dorsal margin abruptly bends upward; knob of aedeagus similar to bird’s head with rounded anterior and small beak-like posterior projection, tip directed caudad……………………………… ***O. macrostachyus*** Baker and Tuttle	Aedeagal shaft with dorsal margin gradually bends upward, with very short pointed anterior projection and very long posterior projection, directed upward distally……………………. ***O. hova*** Meyer
5. Aedeagal knob with posterior projection almost straight, slightly bends dorsad or tip directed caudad……………………………………………………….……. **6**	Aedeagal knob with posterior projection gradually bends ventrad, distally…………………………. **7**
6. Aedeagal shaft bends dorsad forming obtuse angle (*α2* = 120^°^) between shaft and stem axis; knob axis parallel to shaft axis; dorsal margin slightly concave, medially……………. ***O. malawiensis*** Meyer	Aedeagal shaft bends dorsad forming right angle (*α2*) between shaft and stem axis; knob axis forming acute angle (*α1* = 22^°^) to shaft axis; dorsal margin convex, medially…… ***O. sapienticolus*** Gupta
7. Duplex setae on male tarsus I with members very dissimilar in length; female palpus with terminal sensillum about twice or less than twice as long as broad………………………**8**	Duplex setae on male tarsus I with members half to nearly as long as distal member; female palpus with terminal sensillum more than twice as long as broad……………. **9**
8. Aedeagal knob with posterior projection twice as wide as stem; female with terminal sensillum of palpus about twice as long as broad…………………………………………………………. ***O. pemphisi*** Gutierrez	Aedeagal knob with posterior projection slightly wider than width of stem; female with terminal sensillum one and two thirds as long as broad………………………***O. imberbei*** Meyer
9. Tip of posterior projection of aedeagal knob not reaching level of ventral margin of shaft; proximal member of duplex setae of male tarsus I about half as long as distal member; female medioventral striae with lobes……………………………………***O. apohadrus*** Meyer	Tip of posterior projection reaching level of ventral margin of shaft; proximal member of duplex setae of male tarsus I about three-quarters the length of distal member, female medioventral striae devoid of lobes…………………………… ***O. hadrus*** Pritchard and Baker

#### *Key to species of the subgroup *gossypii *(10 species)*

**Table:** 

1. Male with posterior projection of aedeagal knob drawn out in a very slender stylet, directed anterodorsally; female peritreme straight with bulbous end…………………….. ***O. trichardti*** Meyer	Aedeagus with posterior projection not stylet-like but acute and of variable length; female peritreme hooked/retrose distally…………………… **2**
2. Aedeagal knob with posterior projection shorter than stem width……………………………………………… **3**	Aedeagal knob with posterior projection longer than stem width………………………………………………. **4**
3. Palp spinneret of both female and male 4–5 times longer than broad; aedeagal knob with acute anterior projection, dorsal margin of knob slightly sigmoid or concave anteromedially………………………………………………………….…………. ***O. licinus*** Baker and Pritchard	Palp spinneret of both female and male 2–3 times longer than broad; aedeagal knob with blunt or rounded anterior projection, dorsal margin of knob not sigmoid or convex anteromedially…………………………………. ***O. uruma*** Ehara
4. Aedeagal knob with posterior projection evenly curved, directed dorso-caudally; dorsum of knob slightly convex with knob axis forms an acute angle (*α1*) with shaft axis…… ***O. intermedius*** Meyer	Dorsal margin of aedeagal knob strongly sigmoid; if not sigmoid, then rather flat and slightly convex in area of posterior half but knob axis parallel to shaft axis…………………………………………. **5**
5. Dorsal margin of aedeagal knob rather flat and slightly convex in area of posterior half (axis of knob parallel to shaft axis)……………………………………………………………………… **(*litchii* species complex) 6**	Dorsal margin of aedeagal knob strongly angulate…………………………… **7**
6. Female palp spinneret about 2.5 times as long as wide; male tibia I with 9 tactile setae and 4 solenidion, tarsus I with 4 tactile and 3 solenidion behind to proximal duplex, aedeagal knob equal or sub-equal to shaft dorsal margin………………………………… ***O. litchii*** Lo and Ho	Female palp spinneret about 3 times as long as wide; male tibia I with 10 tactile and 3 solenidion, tarsus I with 6 tactile and 1 solenidion behind to proximal duplex, aedeagal knob almost 1.3 times longer than shaft dorsal margin………………………………………………… ***O. taiwanicus*** Tseng
7. Aedeagal knob with posterior projection tip directed caudad/ventrad……………. ***O. gossypii*** Zacher	Aedeagal knob with posterior projection tip directed dorsally………………………………………. **8**
8. Female and male with 4 tactile setae proximal to duplex on tarsus II; dorsal margin of aedeagal shaft short, almost half the length of knob………………………… ***O. matthyssei*** Rimando	Female and male with 3 tactile setae proximal to duplex on tarsus II; dorsal margin of aedeagal shaft almost equal or longer than knob……..…………….. **9**
9. Female medioventral striae without lobes; chromosome numbers n = 4………………… ***O. grewiae*** Meyer	Female with broad, low lobes on medioventral striae between coxae III and IV; chromosome numbers n = 2……………………………***O. randriamasii*** Gutierrez

#### *Key to species of the subgroup *exsiccator* (69 species, excluding two briefly described species)*

**Table:** 

1. Aedeagus with upturned terminal part very long, drawn out in a very slender stylet……………… **2**	Aedeagus with upturned terminal part with small to medium length, without slender stylet like appearance, with/without distal knob or making sigmoidal shape…………………………………..** 3**	
2. Both female and male peritreme straight with bulbous end; male empodium I with proximoventral spur, dorsally directed aedeagus part slender without anterior/posterior projection………………………………………………………………. ***O. flexuosus*** Beer and Lang	Both female and male peritreme strongly hooked distally; male empodium I with free proximoventral hairs, dorsally directed aedeagus part with strong anterior projection or barb basally……………………………… ***O. mcgregori*** (Baker and Pritchard)	
3. In lateral view, aedeagus becoming sigmoid distally or upturned part sigmoidal/sickle shaped, with distal part of upturned portion narrower than basal part; or dorsal projection gradually/abruptly tapering, with distal tip straight or bending anteriorly/posteriorly…………………**4**	In lateral view, aedeagus mostly with distinct knob distally, with distal part of upturned portion wider than basal part; or dorsal projection gradually expanded before curving/tapering posteriorly………………………………………… **22**	
4. Aedeagus with dorsally directed distal part strongly bends anteriorly, bends part straight, almost parallel/subparallel to shaft…………………………………………………….… ***O. manishi*** Gupta	Aedeagus with dorsally directed distal part tapering to form truncate tip, first bends anteriorly then posteriorly or becoming sigmoid distally……………………………. **5**	
5. Aedeagus with dorsally directed distal part almost straight, directed dorsally, gradually or abruptly tapering to form truncate or finger like tip, not forming or barely sigmoid shape; female must with straight peritreme, not hooked………………………………**6**	Aedeagus with dorsally directed distal part forming more or less sigmoid shape, first bends anteriorly then posteriorly, gradually/abruptly bends posteriorly or tip slightly/strongly bends posteriorly/ventrally; female peritreme either hooked or straight…………………………… **7**	
6. Dorsally bent part of aedeagus gradually tapers to a truncate tip………………… ***O. kadarsani*** Ehara	Dorsally bent part of aedeagus abruptly narrowed by sharp inward bend of posterior margin forming sharp bluntly pointed tip…………………………………***O. digitatus*** Davis	
7. Dorsally directed aedeagal projection strongly/weakly sickle shaped, almost basal half initially bent anterior or rather straight then terminal half gradually/abruptly bent posterior with strong/weak obtuse angulation medially, upturned part slender or finger like with blunt/rounded tip directed dorsad or dorsocaudad but never ventrad…………………………………………………………………………………………… **8**	Dorsally directed aedeagal projection not obviously sickle shaped, almost three-fourth part of both or at least anterior margin of dorsal projection straight without medial angulation, gradually bent posterior or almost distal one-third to one-fourth part directed dorsad, dorso-caudad or ventrad……… **12**	
8. Adults with 6 tactile setae on tibia II (almost basal half of upturned aedeagal projection somewhat straight basally, terminal half abruptly bent posterior medially…………………… ***O. waltersi*** Meyer	Adults with 7 tactile setae on tibia II………………………**9**	
9. Dorsally directed aedeagal part longer, strongly angulate medially, with long and slenderer posterior projection………………………………… ***O. grypus*** Pritchard and Baker	Dorsally directed aedeagal part comparatively short, weakly angulate medially, with small posterior projection forming slender, blunt or finger like tip……………………………**10**	
10. Female peritreme straight, without distal hook………………………. ***O. dactyloni*** Smiley and Baker	Female peritreme with short distal hook………………… **11**	
11. Female with striae on genital flap between setae *g*_*1*_ transverse, band of longitudinal striae on pregenital region ending well before level of setae *ag;* male peritreme with distal segment greatly expanded………………………………………………… ***O. oryzae*** Hirst	Striae on genital flap between setae *g*_*1*_ longitudinal, band of longitudinal striae on pregenital region ending almost level with setae *ag;* male peritreme with distal segment slightly expanded………………………………. ***O. zanclopes*** Beard and Walter	
12. Aedeagus with upturned part directed ventrad distally……………………………………………. **13**	Aedeagus with upturned part directed dorsad or dorso-caudad distally………………… **16**	
13. Aedeagus with distal part strongly sigmoid; upturned part recurved distally, forming strong and comparatively thick downturned tip, somewhat similar to knob………………………………… **(*sacchari* species complex)**…………..………….. ***O. sacchari*** McGregor, ***O. saccharinus*** Baker and Pritchard	Aedeagus with distal part shallowly sigmoid; or aedeagus gracefully thin from shaft basis to ventrally bent thin tip, forming goose neck……………………………**14**	
14. Aedeagus gracefully thin from shaft basis to ventrally curved tip forming goose neck; upturned part gradually bent ventrad distally…………………………………. ***O. zeae*** McGregor		Aedeagus with upturned part comparatively thick, straight and abruptly curved ventrad distally; forming weakly sigmoid shape……………………………………….…………………………… **15**
15. Aedeagus with upturned part almost as long as dorsal margin of shaft; male empodium I with medio-dorsal claw about half the length of proximoventral spurs……………………. ***O. duncombei*** Meyer		Aedeagus with upturned part almost half the length of dorsal margin of shaft; male empodium I with medio-dorsal claw about twice as long as proximoventral spurs………………… ***O. triandrae*** Meyer
16. Female peritreme retrose or hooked distally…………………… **17**		Female peritreme straight or with slight distal bend but not hooked, ending in a simple bulb………….………………………………………………………….….. **(*plegas* species complex) 18**
17. Aedeagus with bent part first turns anteriorly then tip abruptly directed dorsad, dorsal margin of shaft upturned forming acute angle with anterior margin of upturned part, male empodium I with proximoventral spur distinctly shorter than empodial claw……………….…………. ***O. neoplegas*** Meyer		Aedeagus with bent part almost straight or gradually turns posteriorly to form caudally/ dorsocaudally directed tip, dorsal margin of shaft upturned forming right/obtuse angle with anterior margin of upturned part, male empodium I with proximoventral spur almost equal or slightly longer than empodial claw…………………………………………………………… ***O. plicarum*** De Leon
18. Dorsally directed aedegal projection very slightly sigmoid, gradually tapered distally, forming dorsally/dorso-caudally directed somewhat acute tip………………………….…… **19**		Dorsally directed aedeagal part comparatively more sigmoid, abruptly bent posterior at distal end forming caudally/dorso-caudally directed acute or blunt/finger like tip………………… **20**
19. Aedeagus with distal sigmoid part forming a right angle with shaft axis……… ***O. sayedi*** Zaher et al		Aedeagus with distal sigmoid part forming an acute angle with shaft axis……………………………………………………………………. ***O. plegas*** Baker and Pritchard
20. Aedeagus with dorsal projection strongly sigmoid distally forming caudally directed truncate tip………………………………………………. ***O. orthius*** Rimando		Aedeagus with dorsal projection slightly sigmoid distally forming dorso-caudally directed somewhat acute/blunt tip……………………………………………………………………………………….. **21**
21. Dorsally directed part nearly straight, ending in a sharply turned dorso-caudally directed tip……………………………………………… ***O. velascoi*** Rimando		Dorsally directed part more curved, not sharply tapered nor abruptly turned tip…. ***O. araneum*** Davis
22. Axis of aedeagal knob parallel or sub-parallel to shaft axis…………………… **23**		Axis of aedeagal knob forming strong or weak angle (*α1*) with shaft axis……………..…………… **30**
23. Aedeagus with stem/neck width about five times longer than knob posterior projection length; female with 8 tactile setae on tibia I (aedeagal knob almost rounded with short spine like downturned posterior projection)………………………………. ***O. gratus*** Tseng		Aedeagus with stem/neck width maximum three times longer than knob posterior projection length; female with 9–10 tactile setae on tibia I…….…………….…... **24**
24. Aedeagal knob with well-developed acute anterior projection (knob similar to bird’s head with beak, posterior projection directed caudad, knob dorsal margin round to acute or convex)………………………………………………………………….. ***O. neotylus*** Zeity and Srinivasa		Aedeagal knob with rounded anterior projection, either absent or well-developed………………… **25**
25. Aedeagal knob with anterior projection almost absent or narrowly rounded (knob stem almost 4 to 5 times wider than length of knob anterior projection)…………………………………….. ***O. modestus*** (Banks)		Aedeagal knob with anterior projection well-developed or broadly rounded………………………. **26**
26. Aedeagal knob with posterior projection terminating in caudally directed blunt finger-like tip, knob dorsal margin almost straight or narrowly rounded……………………………….….. ***O. pallus*** Beard		Aedeagal knob with posterior projection terminating in ventrally/caudally directed acute tip, knob dorsal margin broadly rounded………………………………….…. **(*afrasiaticus* species complex) 27**
27. Aedeagal knob with posterior projection about four times longer than anterior projection; male palp spinneret almost 2.5 times longer than wide…………………….……………….. ***O. aquilinus*** Meyer		Aedeagal knob with posterior projection about ≤ 3.5 times longer than anterior projection; male palp spinneret almost two times longer than wide…………………….…………………………………. **28**
28. Posterior projection of aedeagal knob directed caudad distally…………….. ***O. menezesi*** Flechtmann		Posterior projection of aedeagal knob deflexed, directed ventrad distally………………………….. **29**
29. Posterior projection of aedeagal knob ending in attenuate tip…………… ***O. keiferi*** Tuttle and Baker		Posterior projection of aedeagal knob acute, without attenuate tip……. ***O. afrasiaticus*** (McGregor)
30. Aedeagal knob about as long as or longer than shaft dorsal margin, with posterior projection at least four times longer than anterior projection (posterior projection must rounded, except *O. martensis*; female with nine tactile setae on tibia I)…………………………. **31**		Aedeagal knob comparatively short or virtually non-existent, maximum three-fourths the length of shaft dorsal margin; or with posterior projection shorter, equal, subequal or rarely longer than anterior projection………………………… **36**
31. Adults with 6 tactile setae on tibia II……………………. ***O. nasutus*** Meyer		Adults with 7 tactile setae on tibia II………………… **32**
32. Aedeagal knob with posterior projection straight and directed dorsad………………… **33**		Aedeagal knob with posterior projection gradually or abruptly curving ventrad………… **34**
33. Aedeagal knob with a needle like posterior projection; shaft axis forming an angle (*α1*) of about 115^°^ with knob axis………………………………… ***O. leandrianae*** Gutierrez		Aedeagal knob with posterior projection gradually tapering to a sharp tip; shaft axis forming an angle (*α1*) more than 120^°^ with knob axis…………………… ***O. bessardi*** Gutierrez
34. Aedeagal knob with posterior projection as long as or shorter than stem………………………………………………………… ***O. calicicola*** Knihinicki and Flechtmann		Aedeagal knob with posterior projection distinctly longer than stem…………….………………… **35**
35. Aedeagal knob about 4 times as long as its stem width, posterior projection of knob basally directed dorsad with tip abruptly turned ventrad or hooked; female with palp spinneret about as long as wide……………………………………………………………………………… ***O. martensis*** Meyer		Aedeagal knob about 3 times as long as its stem width, posterior projection of knob gradually turned ventrad or tip straight; female with palp spinneret about 1.3 times as long as wide………………………………………………………………………………. ***O. barbatae*** Meyer
36. Aeeagal knob with posterior projection distinctly longer or ≥ 1.5 times longer than stem height…** 37**		Aeeagal knob with posterior projection distinctly shorter, equal, sub-equal or slightly longer than stem height………………………………………………………………………………………….. **42**
37. Aedeagal knob with small acute anterior projection.…………………………**38**		Aedeagal knob with broadly/narrowly rounded anterior projection…………………**39**
38. Aedeagal knob with dorsal margin undulating, slightly concave medially, posterior projection tip directed dorsad/dorso-caudad; male/female with 8 and 6 tactile setae on tibia I and II, respectively……………………… ***O. oenotherae*** Smiley and Baker		Aedeagal knob with dorsal margin convex, not undulating, posterior projection tip directed ventro-caudad; male/female with 9 and 7 tactile setae on tibia I and II, respectively… ***O. castrensis*** Meyer
39. Aedeagal knob with posterior projection directed ventro-caudad, tip bending ventrad; knob length ≥ 3 times longer than stem width…………..…………………… **40**		Aedeagal knob with posterior projection directed dorsad or dorso-caudad, tip not bending ventrad; knob length < 3 times longer than stem width………**41**
40. Aedeagal knob with posterior projection > 3 times longer than anterior projection, posterior projection about ≥ 2.5 times longer than stem height; knob axis forming about a 45° angle (*α1*) with shaft axis………………………………………. ***O. gramineus*** (McGregor)		Aedeagal knob with posterior projection < 2 times longer than anterior projection; posterior projection about < 2.5 times longer than stem height; knob axis forming obviously less than a 45° angle (*α1*) with shaft axis………………………***O. stickneyi*** (McGregor)
41. Aedeagal knob with posterior projection ending in acute tip, knob length about two times the stem width, posterior projection < 3 times longer than anterior projection……..… ***O. exsiccator*** (Zehntner)		Aedeagal knob with posterior projection ending in blunt tip, knob length about > 2.5 times the stem width, posterior projection ~ 3.5 times longer than anterior projection… ***O. turbelli*** Beard and Walter
42. Female with dorsal hysterosomal striae longitudinal, irregular longitudinal or oblique between *e*_*1*_*-e*_*1*_ setae …………… **43**		Female with dorsal hysterosomal striae transverse between *e*_*1*_*-e*_*1*_ setae, longitudinal or oblique striae between or posterior to *f*_*1*_ setae………………**44**
43. Aedeagal knob > 3 times longer than stem width; female peritreme hooked, distally; palp spinneret longer, about > 2 times longer than wide (male and female); male empodium I with 3 pairs of proximoventral hairs………………………………………… ***O. andrei*** Gutierrez		Aedeagal knob ≤ 2 times longer than stem width; female peritreme straight, not hooked; palp spinneret shorter, about as long as wide (male and female); male empodium I with proximoventral spur……………………………………………… ***O. indicus*** (Hirst)
44. Female tibia I with 8 tactile setae……………………………**45**		Female tibia I with 9 tactile setae…………………………… **46**
45. Female tibia II with 6 tactile setae; aedeagus with shaft dorsal margin about two times long as knob length……………………………………………………………………………………… ***O. nelensis*** Meyer		Female tibia II with seven tactile setae; aedeagus with shaft dorsal margin ≥ 2.5 times long as knob length…………..………………………………………………………………… ***O. rusticus*** Meyer
46. Female with peritreme U-shaped or hooked distally……………………**47**		Female with peritreme straight distally, ending simply or in a bulb……………………………**49**
47. Aedeagal knob with anterior projection rounded; male peritreme straight, ends simply; female palp spinneret about twice longer than wide………………………………………. ***O. neopratensis*** Meyer		Aedeagal knob with anterior projection acute or weakly developed; male peritreme hooked distally; female palp spinneret about as long as or slightly longer than wide………………………………**48**
48. Aedeagal knob with anterior projection acute, posterior projection about as long as anterior projection; female palp spinneret about as long as wide…………………………… ***O. chazeaui*** Gutierrez		Aedeagal knob with anterior projection almost absent, weakly developed or narrowly rounded, posterior projection ≥ 2 times longer than anterior projection; female palp spinneret slightly longer than wide…………………………………………………………………..………… ***O. rubicundus*** Ehara
49. Aedeagal knob with dorsal margin shallowly concave; male with palp spinneret slightly longer than wide; female palp spinneret wider than long…………………………………… ***O. simus*** Baker and Pritchard		Aedeagal knob with dorsal margin convex or straight…………………….…………………**50**
50. Female with 6 tactile setae on tibia II……………………………….. **51**		Female with 7 tactile setae on tibia II……………………………………**52**
51. Aedeagal knob with dorsal margin almost straight, posterior projection about two-fifth as long as stem height or stem width; male with leg I empodial claw longer than spur, male/female palp spinneret ≥ 2 times longer than wide; female with 4 tactile setae proximal to duplex on tarsus II………………………………………………………… ***O. andropogonearum*** Gutierrez		Aedeagal knob with dorsal margin slightly convex medially or undulating, posterior projection about half as long as stem height or stem width; male with leg I empodial claw shorter than spur, male/female palp spinneret about as long as or slightly longer than wide; female with 3 tactile setae proximal to duplex on tarsus II……….……………… ***O. ephamnus*** Beard and Walter
52. Male empodium I with 3 pairs of proximoventral hairs (aedeagal knob less than half as long as shaft dorsal margin; female/male with palp terminal sensillum > 1.5 times longer than wide)…………………………………………… ***O. grastis*** Meyer		Male empodium I with proximoventral hairs modified to form single or paired spur…………**53**
53. Aedeagal knob like rounded protuberance, with almost no anterior or posterior projection, if present then both projections and dorsal margin rounded/convex medially or only posterior projection acute/acutely angulate (male with only empodium I with proximoventral spurs and empodium II with hairs) ……………………………** (*tylus* species complex) 54**		Aedeagal knob not like rounded protuberance, like beak/bird's head or shape variable, with distinct anterior and/or posterior projection, if any projection nearly absent then it acutely angulate and dorsal margin either straight, convex or rounded…………………………… **56**
54. Aedeagal knob with posterior projection nearly absent, both projections and dorsal margin like rounded protuberance; female palp spinneret about as long as wide………………. ***O. etiennei*** Gutierrez		Aedeagal knob with anterior projection nearly absent or narrowly rounded/convex, posterior projection slightly acute/acutely angulate, dorsal margin convex medially or obtusely angulate; female palp spinneret slightly or 1.5 times longer than wide………………………………**55**
55. Aedeagus with shaft dorsal margin forming almost right angle to anterior margin of upturned part; male empodium I with proximoventral spurs longer than dorsal claw; female palp spinneret slightly longer than wide………………………………………………………. ***O. tylus*** Baker and Pritchard		Aedeagus with shaft dorsal margin forming almost acute angle to anterior margin of upturned part; male empodium I with proximoventral spurs as long as dorsal claw; female palp spinneret about 1.5 times longer than wide……………………………………….. ***O. senegalensis*** Gutierrez and Etienne
56. Aedeagal knob with anterior projection virtually absent (dorsal margin almost straight, posterior projection tip directed dorsad/dorso-caudad, not ventrad)..…………………………………………… **57**		Aedeagal knob with distinct anterior projection, dorsal margin broadly/narrowly rounded convex medially or rarely straight, posterior projection tip directed dorsad, dorso-caudad or ventrad……….. **59**
57. Female with palp spinneret about as long as wide…………. ***O. mexicanus*** (McGregor and Ortega)		Female with palp spinneret about 1.8 to 2 times longer than wide…………………………………. **58**
58. Male empodium I and II both with proximoventral spurs; aedeagal knob with stem comparatively longer, stem height about as long as or longer than knob length and about half as long as shaft dorsal margin and shaft width………………………………………………………… ***O. pennisetum*** Meyer		Male empodium I with proximoventral spurs and empodium II with proximoventral hairs; aedeagal knob with stem comparatively shorter, stem height about half as long as knob length and one-fourth as long as shaft dorsal margin and shaft width……………………….. ***O. obliquus*** Ehara and Masaki
59. Aedeagal shaft forming obtuse angle (*α2*) with axis of bent part or shaft turns dorsad at obtuse angle…………………………………………………………….. ***O. formosanus*** Lo		Aedeagal shaft forming right/acute angle (*α2*) with axis of bent part or shaft turns dorsad at right/acute angle…………… **60**
60. Aedeagal knob maximum 1.3 times longer than stem width (both anterior and posterior projections of knob with similar length and acute, knob dorsal margin convex/obtusely angulate; only male empodium I with proximoventral spur, empodial claw obviously longer than spur)……………………………………………………***O. campestris*** Meyer		Aedeagal knob ≥ 1.5 times longer than stem width (knob anterior projection rounded/acute)…….. **61**
61. Both male and female with short peritreme, ends simply without terminal bulb or peritreme inconspicuous in male (aedeagal knob with anterior projection and dorsal margin broadly rounded, posterior projection acute, directed dorsad/dorso-caudad; only male empodium I with proximoventral spur, longer than empodial claw)…………………… ***O. hortulanus*** Meyer		Female and/or male peritreme of normal length, with/without bulbous end.……………………….. **62**
62. Male empodium I and II both with paired proximoventral spurs………………………**63**		Male empodium I only with proximoventral spur and empodium II with proximoventral hairs………………………………… (***pratensis* species complex) 64**
63. Male empodium II with proximoventral claw distinctly shorter than dorsal empodial claw and withtwo fine dorsal hairs; aedeagus with shaft dorsal margin as long as the shaft greatest width; knob with posterior projection shorter than anterior projection………………………… ***O. ocellatus*** Meyer		Male empodium II with proximoventral claw distinctly longer than dorsal empodial claw and without fine dorsal hairs; aedeagus with shaft dorsal margin distinctly longer than shaft greatest width; knob with posterior projection longer than anterior projection …… ***O. washingtoniae*** Mushtaq et al
64. Female palp spinneret about 2.5 times as long as broad (male palp spinneret about two times as long as broad; knob about fifth the length of shaft dorsal margin; only empodium I with proximoventral spur, longer than main claw)…………………………………………………………………….. ***O. krantzi*** Zaher et al		Female palp spinneret ≤ 2 times as long as broad……………………**65**
65. Posterior projection of aedeagal knob truncated caudally (knob dorsal margin with convex/angulated medially…………………………………………………………………………………………. ***O. shinkajii*** Ehara		Posterior projection of aedeagal knob acute caudally …………………… **66**
66. Knob length almost equal to the height of knob stem; posterior projection of aedeagal knob > 4 times longer than anterior projection, and about half as long as stem height…………… ***O. virens*** Gutierrez		Knob length distinctly greater than the height of knob stem; posterior projection of aedeagal knob < 2 times longer than anterior projection, and about equal or slightly longer than stem height……… **67**
67. Posterior projection of aedeagal knob about sub-equal or slightly longer than anterior projection, shaft dorsal margin maximum 2.5 the length of knob…………………………. ***O. bahaensis* sp. nov**		Posterior projection of aedeagal knob about 1.5 times longer than anterior projection, shaft dorsal margin about three times longer than knob length…………… ***O. pratensis*** (Banks)

*O. anneke Baker and Pritchard could not include in key to species of the subgroup *exsiccator* due to the unavailability of morphological information regarding the number of tactile setae on tibia II in females.

*O. saccharoides Baker and Tuttle could not include in key to species of the subgroup *exsiccator* due to the unavailability of male morphological information about proximoventral hair/spur on empodium II.

## Discussion

A diagnostic key has been erected for distinguishing the subgenera, species groups, and species subgroups of the genus *Oligonychus*^5^. In the present study, five dichotomous keys are developed using male/female morphological characters to identify the world *Oligonychus* species belonging to five *Reckiella* subgroups (*biharensis*, *exsiccator*, *gossypii*, *iseilemae*, and *pritchardi*), except the subgroup *comptus* that having only one species^5^. However, five *Oligonychus* (*Reckiella*) species, namely, *O. annonicus* (McGregor)*, O. anneke, O. bruneri* (Livschitz)*, O. stenoperitrematus* (Ugarov and Nikolskii)*,* and *O. saccharoides* could not be included in species-level keys due to the lack of required key morphological information in the published literature^25–29^. Among them, *O. annonicus* and *O. stenoperitrematus* did not even assign to any species group due to the unavailability of required female morphological characters. Also, *O. bruneri* was only assigned to the level of species group *exsiccator*^5^.

The subgroup *exsiccator* is the most diverse grouping *in Reckiella*^5^. In this subgroup, we found some intraspecific variations in few species, namely, *O. formosanus, O. indicus, O. modestus, O. pennisetum*, and *O. pratensis.* In some specimens of *O. pennisetum,* the female striae pattern was observed as irregular instead of having transverse between setae *e*_*1*_*-e*_*1*_^8^. Also, the shape of male aedeagus in one reported population of *O. indicus* is totally different (knob dorsal margin concave, with distinct anterior projection)^4^ from original or other described populations (knob dorsal margin straight, with almost no anterior projection)^16,30^. Likewise, one aedeagal illustration of *O. formosanus* looks somewhat different (shaft axis forming a nearly right angle to the axis of the bent part^31^, as compared to other reported populations^32,33^. In *O. modestus,* the knob dorsal margin shape was also variable from convex/rounded^4,11^ to almost straight^34,35^. In *O. pratensis,* the shape of aedeagus (specifically aedeagal knob) was found to be variable among various populations, reported from different geographical localities^4,8,10,11,27,34,36–39^. Indeed, the Chinese population of *O. pratensis* was detected as a cryptic species that even did not belong to the subgroup *exsiccator*^5,12^. Furthermore, Meyer^10^ synonymized *O. monsarrati* Gutierrez with *O. grypus* and also agreed by the later authors^5^. However, still, *O. monsarrati* has been considered a valid species^2,3,40^, but no study proved/discussed its validity.

Moreover, some *Oligonychus* (*Reckiella*) species viz. *O. aquilinus*, *O. afrasiaticus*, *O. keiferi*, and *O. menezesi* represent the *afrasiaticus* complex; *O. litchii* and *O. taiwanicus* represent the *litchii* complex; *O. araneum*, *O. orthius*, *O. plegas*, *O. sayedi*, and *O. velascoi* representing the *plegas* complex; *O. etiennei*, *O. senegalensis* and *O. tylus* represent the *tylus* complex; *O. sacchari* and *O. saccharinus* represent the *sacchari* complex; and, *O. krantzi*, *O. pratensis*, *O. shinkajii*, and *O. virens* represent the *pratensis* complex^5,10,12,41^ are morphologically very closed to each other. However, some of these species are distinguished in the present study using some characteristics, e.g., the number of tactile/sensory setae on male tibia I, tactile setae behind to proximal duplex on male tarsus I, and the shape of male aedeagus. Still, some species (*O. pratensis*, *O. sacchari*, *O. saccharinus*) are difficult to differentiate using their available morphological information because key characters of aedeagus are variable in their expression among/within reported populations^4,8,10–12,15,27,34,36–39,41^. Therefore, we brought the specimens of *O. pratensis* from USA (the country of its type locality) and observed the key diagnostic character of male aedeagus to differentiate between *O. pratensis* (Fig. 6) and the new species *O. bahaensis* sp. nov. (Fig. 4C,D).

In conclusion, we developed five diagnostic keys based on the published morphotaxonomic literature to facilitate the identification of world *Oligonychus* (*Reckiella*) species. We suggest using molecular data with morphology to validate the identification of any newly described or closely related *Oligonychus* species. Integrative taxonomic approaches are crucial to resolving the issues of species complexes in the subgenus *Reckiella*.

## Data Availability

All data generated or analysed during this study are included in this published article. The type specimens of new species are available at the King Saud University Museum of Arthropods (Acarology section), Saudi Arabia, and can be obtained from the corresponding author on reasonable request.

## References

[1] Hoy MA (2011). Agricultural acarology: Introduction to integrated mite management.

[2] Migeon, A. & Dorkeld, F. *Spider Mites Web: A comprehensive database for the Tetranychidae*, http://www1.montpellier.inra.fr/CBGP/spmweb (2022).

[3] Bolland HR, Gutierrez J, Flechtmann CHW (1998). World catalogue of the spider mite family (Acari: Tetranychidae).

[4] Jeppson LR, Keifer HH, Baker EW (1975). Mites injurious to economic plants.

[5] Mushtaq HMS, Alatawi FJ, Kamran M, Flechtmann CHW (2021). The genus *Oligonychus* Berlese (Acari, Prostigmata, Tetranychidae): Taxonomic assessment and a key to subgenera, species groups, and subgroups. Zookeys.

[6] Mushtaq HMS, Kamran M, Alatawi FJ (2022). New species, new records, and re-descriptions of two species of the genus *Oligonychus* Berlese (Acari: Prostigmata: Tetranychidae) from Saudi Arabia. Syst. Appl. Acarol..

[7] Mushtaq HMS, Kamran M, Saleh AA, Alatawi FJ (2023). Evidence for reconsidering the taxonomic status of closely related *Oligonychus* species in punicae complex (Acari: Prostigmata: Tetranychidae). Insects.

[8] Meyer, M. K. P. S. *African Tetranychidae (Acari: Prostigmata)—With reference to the world genera, entomology memoir*. (Department of Agriculture and Water Supply, 1987).

[9] Ben-David, T. Molecular Characterization of Israel's Spider Mites (*Acari: Tetranychidae*). Ph.D. thesis, Hebrew University, (2008).

[10] Meyer, M. K. P. S. *A revision of the Tetranychidae of Africa (Acari) with a key to the genera of the world, Entomology Memoir*. (Department of Agricultural Technical Services, 1974).

[11] Pritchard, A. E. & Baker, E. W. *A revision of the spider mite family Tetranychidae*. (Pacific Coast Entomological Society, 1955).

[12] Li J, Yi T-C, Guo J-J, Jin D-C (2018). Ontogenetic development and redescription of *Oligonychus pratensis* (Banks, 1912) (Acari: Tetranychidae). Zootaxa.

[13] Rimando LC (1962). The tetranychoid mites of the Philippines. Univ. Philipp. Coll. Agric. Tech. Bull..

[14] Zaher MA, Gomaa EA, El-Enany MA (1982). Spider mites of Egypt (Acari: Tetranychidae). Int. J. Acarol..

[15] Beard JJ, Walter DE, Allsopp PG (2003). Spider mites of sugarcane in Australia: A review of grass-feeding *Oligonychus* Berlese (Acari: Prostigmata: Tetranychidae). Aust. J. Entomol..

[16] Zeity, M. Tetranychid mite fauna of major agroecosystems in Karnataka and some aspects of molecular characterization of selected genera of Spider. Ph.D. thesis, University of Agricultural Sciences, (2015).

[17] Arabuli T, Gotoh T (2018). A new species of spider mite, *Oligonychus neocastaneae* sp.nov. (Acari: Tetranychidae), from Japan. Zootaxa.

[18] Ganjisaffar F, Perring TM (2015). Prey stage preference and functional response of the predatory mite *Galendromus flumenis* to *Oligonychus pratensis*. Biol. Control.

[19] Lindquist, E. E. in *Spider mites: Their biology, natural enemies and control* (eds Helle, W. & Sabelis, M. W.) Ch. 1.1, 3–28 (Elsevier, 1985).

[20] Grandjean F (1939). Les segments post-larvaires de l'hystérosoma chez les Oribates (Acariens). Bull. Soc. Zool. Fr..

[21] Grandjean, F. Les Enarthronota (Acariens). 1e serie. *Annales des Sciences Naturelles-Zoologie et Biologie Animale* **8**, 213–248 (1947).

[22] Mushtaq, H. M. S. Morphometric, molecular, and webbing behavioral studies on some spider mite species of the genus *Oligonychus Berlese* (Acari: Tetranychidae) in Saudi Arabia. Ph.D. thesis, King Saud University, (2022).

[23] Ben-David T, Melamed S, Gerson U, Morin S (2007). ITS2 sequences as barcodes for identifying and analyzing spider mites (Acari: Tetranychidae). Exp. Appl. Acarol..

[24] Alatawi FJ, Kamran M (2018). Spider mites (Acari: Tetranychidae) of Saudi Arabia: Two new species, new records and a key to all known species. J. Nat. Hist..

[25] Ugarov AA, Nikolskii VV (1937). Systematic study of spider mites from Central Asia. Trudy Uzb. Opytn. Stantsii Zashchity Rastenii (Tashkent).

[26] McGregor EA (1955). Notes on spider mites (Tetranychidae) of Ecuador. Rev. Ecuat. Entomol. Parasitol..

[27] Baker EW, Pritchard AE (1962). Arañas rojas de América Central (Acarina: Tetranychidae). Rev. Soc. Mex. Hist. Nat..

[28] Livschitz, I. S. & Salinas, C. A. *Preliminares acerca de los ácaros tetránicos de Cuba*. (Centro Nacional Fitosanitário, 1968).

[29] Baker EW, Tuttle DM (1972). New species and further notes on the Tetranychoidea mostly from the Southwestern United States (Acarina: Tetranychidae and Tenuipalpidae). Smithson. Contrib. Zool..

[30] Hirst, S. in *Proceedings of the Zoological Society of London.* 971–1000.

[31] Ehara S, Gotoh T (2007). Five species of spider mites (Acari: Prostigmata: Tetranychidae) from Japan with descriptions of two new species. Zootaxa.

[32] Lo PKC (1969). Tetranychoid mites infesting special crops in Taiwan. Bull. Sun Yatsen Cult. Found..

[33] Lo PKC, Ho CC (1989). The spider mite family Tetranychidae in Taiwan I. The genus Oligonychus. J. Taiwan Mus..

[34] Baker, E. W. & Tuttle, D. M. *A guide to the spider mites (Tetranychidae) of the United States*. (Indira Publishing House, 1994).

[35] Beglyarov GA, Mitrofanov VI (1973). New species of the genus *Oligonychus* and *Tetranychus* from Vladivostok area. Biol. Revue Zool..

[36] McGregor, E. A. in *Proceedings of the Entomological Society of Washington.* 247–256.

[37] Meyer MKP, Ryke PAJ (1959). A revision of the spider mites (Acarina: Tetranychidae) of South Africa with descriptions of a new genus and new species. J. Entomol. Soc. South. Afr..

[38] Estebanes GML, Baker EW (1968). Arañas rojas de Mexico (Acarina: Tetranychidae). Anal. Esc. Nac. Cienc. Biol..

[39] Tuttle DM, Baker EW, Abbatiello M (1976). Spider mites of Mexico (Acarina: Tetranychidae). Int. J. Acarol..

[40] Migeon A (2015). The Jean Gutierrez spider mite collection. Zookeys.

[41] Khanjani M, Khanjani M, Seeman OD (2018). The spider mites of the genus *Oligonychus* Berlese (Acari: Tetranychidae) from Iran. Syst. Appl. Acarol..

